# The Risk of Tuberculosis Infection in Non-dialysis Chronic Kidney Disease Patients

**DOI:** 10.3389/fmed.2021.715010

**Published:** 2021-08-13

**Authors:** Chia-Hsiang Li, Hung-Jen Chen, Wei-Chun Chen, Chih-Yen Tu, Te-Chun Hsia, Wu-Huei Hsu, Chiz-Tzung Chang, Chiu-Ching Huang, Da-Tian Bau, Che-Yi Chou

**Affiliations:** ^1^Graduate Institute of Biomedical Science, China Medical University, Taichung, Taiwan; ^2^School of Medicine, China Medical University, Taichung, Taiwan; ^3^Division of Pulmonary and Critical Care Medicine, Department of Internal Medicine, China Medical University Hospital, Taichung, Taiwan; ^4^School of Nursing, China Medical University, Taichung, Taiwan; ^5^Division of Nephrology and Kidney, Department of Internal Medicine, China Medical University Hospital, Taichung, Taiwan; ^6^Terry Fox Cancer Research Laboratory, Department of Medical Research, China Medical University Hospital, Taichung, Taiwan; ^7^Department of Bioinformatics and Medical Engineering, Asia University, Taichung, Taiwan; ^8^Division of Nephrology, Asia University Hospital, Taichung, Taiwan; ^9^Department of Post-baccalaureate Veterinary Medicine, Asia University, Taichung, Taiwan

**Keywords:** tuberculosis, chronic kidney disease, body mass index, estimate GFR, non-dialysis CKD

## Abstract

**Background:** Patients with chronic kidney disease (CKD) receiving maintenance renal replacement therapy are at higher risk of tuberculosis (TB) infection. The risk of TB infection in CKD patients not receiving dialysis is unknown.

**Aim:** We conduct this study to test the hypothesis that TB infection is negatively correlated to renal function.

**Design:** Non-dialysis CKD stage 1–5 patients, admitted in China Medical University Hospital from January of 2003 to May of 2014, were enrolled in this study and were prospectively followed up to the diagnosis of TB, death, loss to follow-up, or December 2014. The risk factors of TB infection were analyzed using competing-risks regression analysis with time-varying covariates. The initiation of dialysis and patients' death were considered as competing events. Patients' estimated glomerular filtration rate (eGFR) and body mass index (BMI) were recorded at enrollment.

**Results:** They were followed-up for a median duration of 1.4 years. Of the 7221 patients, TB infection was identified in 114 patients. Higher eGFR was associated with lower risk of TB infection (*P* < 0.01). The adjusted subdistribution hazard ratio (aSHR) was 0.82 [95% confidence interval (CI), 0.72 to 0.94] for every 5 ml/min/1.73 m^2^ increase in eGFR. In addition, higher BMI (*p* = 0.01) was associated with a lower risk of TB infection and the aSHR was 0.91 (95% CI, 0.85 to 0.98) for every 1 kg/m^2^ increase in BMI.

**Conclusion:** Renal function and body mass index are independently associated with the risk of tuberculosis infection in patients with chronic kidney disease not receiving dialysis.

## Introduction

Tuberculosis (TB) is a communicable disease caused by bacillus Mycobacterium tuberculosis, typically affecting the lungs. Despite improvements in the public health structure and system, TB remains a serious public health problem worldwide. The risk of TB is high among immunocompromised individuals, especially in patients with human immunodeficiency virus (HIV) infection. The mortality rate of TB infection is higher if left untreated. In a systemic review of patients with pulmonary TB, the 10-year mortality rate was 70% (1). According to the global tuberculosis report 2015, the estimated global incidence of TB was 10.0 million, and TB is responsible for 1.3 million deaths among HIV-negative people in 2017 (2). There were 12,338 TB cases and 626 TB-related deaths in Taiwain in 2012 (3).

Chronic kidney disease (CKD) is associated with an increased risk of all-cause mortality, cardiovascular events, and hospitalization (4). Estimated glomerular filtration rate (eGFR) is strongly associated with the increased risk of all-cause mortality, cardiovascular events, and hospitalization. The prevalence of all stages of CKD in Taiwan is 12% (95% CI 11·7–12·3), and only 3.5% of patients are aware of their CKD (5). Patients with CKD stage 5 on dialysis had a higher risk of TB than the general population, and these patients are more likely to develop extra-pulmonary TB, especially TB peritonitis and lymphadenitis (6). The incidence of TB was 4.39 per 1000 person-year in CKD patients (7). Refugees with CKD in the Unite State had a higher rate of TB disease (1.9 vs. 0.8% of the general population) (8). None of the previous studies explore the association of renal function and risk of TB infection. We explored the association of eGFR and TB in non-dialysis CKD patients in this cohort study. Competing risks analysis was used because eGFR is associate with increase all-cause mortality.

## Methods

This is a prospective single-center observation study. All patients in the outpatient-based CKD program of China Medical University Hospital (CMUH) from June 2003 to May 2014 were enrolled in this study as per clinicians' discretion. The CKD program included patients with CKD stages 1–5 without dialysis. The nephrologists made the diagnosis of CKD. The CKD program supported by National Health Insurance Taiwan includes clinical endocrinologists, nephrologists, CKD nurses, dietitians, and social workers. The purpose of the CKD program is to monitor the progress of CKD patients. As part of this program, laboratory measurements and CKD education are performed at least four times a year. Basic information, including age, gender, smoking history, and body mass index (BMI), was collected at enrollment. The basal eGFR estimated by the Chronic Kidney Disease Epidemiology Collaboration (CKD-EPI) equation (9) was used in the analysis. CKD was defined as an eGFR <60 ml/min/1.73 m^2^ or markers for kidney damage for three months, and the eGFR and albuminuria defined the CKD stages (10). Hemoglobin, platelet, blood urea nitrogen (BUN), uric acid, fasting blood glucose (FBG), sodium, potassium, calcium, phosphate, albumin, cholesterol, and triglyceride were obtained within three months. All patients were followed from the date of enrollment to the detection of first TB infection, renal replacement therapy (RRT) including hemodialysis, peritoneal dialysis, kidney transplant, loss to follow-up, death, or December 2014. Internal review board approval (DMR 99-IRB-301) was obtained, and the need for informed consent was waived.

A definitive diagnosis of tuberculosis was positive culture for the Mycobacterium tuberculosis complex (*N* = 81), and a presumptive diagnosis of tuberculosis was defined as (1) positive sputum smear examination (positive for acid-fast stain), *N* = 0; (2) radiographic abnormality consistent with pulmonary TB and no response to current board spectrum antibiotics that clinician decided to treat with a full course anti-tuberculosis chemotherapy, *N* = 14; (3) lymphocyte rich exudative pleural effusion adenosine deaminase (ADA) level more than 40 U/L (11), *N* = 5; and (4) histological finding consistent with TB infection (*N* = 14). The pulmonary TB case was a patient with a TB infection involving lung parenchyma. An extra-pulmonary case was a patient with TB infection with involvement of organs other than the lung. Patients who developed both pulmonary and extra-pulmonary tuberculosis were defined as having concomitant TB infection. This TB infection can refer to active TB infection because all patients received standard anti-tuberculosis therapy after the diagnosis.

Patients' comorbidities were recorded at enrollment. Various comorbidities were as follows: Cardiovascular disease, which was defined as a positive exercise test, angiographic findings of at least one stenosis of >50%, or positive findings on scintigraphy. Diabetes was defined as insulin use, a hypoglycemic agent, or a fasting plasma glucose level of 126 mg/dl or more (12). Hypertension was defined as taking antihypertensives or having a systolic BP reading >140 mmHg or a diastolic BP reading >90 mmHg (13). Liver cirrhosis, defined as abnormal image finding with specific clinical laboratory date and manifestation (14). In addition, any form of cancer reported by the patient or detected from medical records was also recorded at enrollment.

### Statistical Analysis

Data were reported as mean ± standard deviation, median (interquartile range), or frequency (percentage) as appropriate. We tested the distribution of continuous variables with skewness and kurtosis tests. Student's *t*-test was applied for parametric variables, Kolmogorov-Smirnov test for non-parametric variables, and chi-square test for categorical variables. A subdistribution hazard ratio (SHR) and 95% confidence interval (95% CI) of the SHR of possible confounders were calculated using univariate competing-risks regression analysis followed by multivariate competing-risks regression (15). TB infection was defined as the primary event in the competing-risks regression, and the competing events include death or loss to follow-up. All analyses were performed using Stata software, version 12 SE (Stata Corp., College Station, TX). *P*-value of <0.05 was considered to represent statistically significant differences.

## Results

In this study, 7,221 non-dialysis CKD stage 1–5 patients were included in the analysis (Table 1) and were followed up for a median period of 1.4 years (interquartile range: 0.7–2.4). Of these, 114 (1.6%) patients had TB infection, and 610 (14.6%) patients were lost to follow-up. The mortality rate was 4.1 per 100 patient-years, and the rate of patients requiring renal replacement therapy (RRT; including hemodialysis, peritoneal dialysis, and kidney transplant) was 12.1 per 100 patient-years. At enrollment, patients' basal eGFR was 35 ± 26 ml/min/1.73 m^2^ and the distribution of patients among various CKD stages were as follows: stage 1, 3.9%; stage 2, 9.2%; stage 3, 37.5%; stage 4, 22%; and stage 5 27.5%. The most common cause of kidney disease was diabetes (38.4%), followed by chronic glomerulonephritis (33%) and hypertension (16.2%). Diabetes was also the most common comorbidity in CKD patients. Basal laboratory parameters' values were recorded in Table 1.

**Table 1:** Clinical characteristics of patients with and without tuberculosis (TB).

**Characteristics**	**Total**		**TB (–)**		**TB (+)**		***p***
	***N*** **=** **7221**		***N*** **=** **7107**		***N*** **=** **114**		
Age (year)	69	±14	69	±14	74	±11	<0.01
Male gender *n* (%)	4153	57.5	4080	57.4	73	64	0.16
BMI (kg/m^2^)	24.4	±4.1	24.5	±2.8	23.8	±2.8	<0.01
eGFR (ml/min/1.73m^2^)	35	±26	35	±26	25	±18	<0.01
Smoking *n* (%)	1189	16.5	1173	16.5	16	14	0.48
**Stage** ***n*** **(%)**							
1	280	3.9	280	4	0	0	0.02
2	664	9.2	661	9.3	3	2.6	<0.01
3	2704	37.5	2668	37.5	36	31.6	0.27
4	1585	22	1555	21.9	30	26.3	0.29
5	1988	27.5	1943	27.3	45	39.5	<0.01
**Causes of kidney disease** ***n*** **(%)**							
Diabetes	2769	38.4	2719	38.3	50	43.9	0.15
Chronic glomerulonephritis	2381	33.0	2341	32.9	40	35.1	0.6
Hypertension	1173	16.2	1161	16.3	12	10.5	0.07
**Comorbidity** ***n*** **(%)**							
Cardiovascular disease	494	6.8	481	6.8	13	10.2	0.13
Diabetes	3094	42.9	3041	42.8	53	46.5	0.43
Cancer	354	4.9	350	4.9	4	3.1	0.35
Liver cirrhosis	222	3.1	215	3	7	6.1	0.06
Hemoglobin (g/dl)	9.6	±2.3	9.6	±2.3	10	±2.8	0.66
Platelet (1000/μl)	195.9	±74.8	196	±75	193	±83	0.65
BUN (mg/dl)	44	±31	44	±31	51	±26	0.03
Creatinine (mg/dl)	3.1	±2.9	3.1	±2.9	3.5	±2.3	0.09
Uric acid (mg/dl)	7.5	±1.9	7	±2	8	±2	0.37
Sodium (meq/L)	138	±3	137.9	±3.4	138.6	±3.4	0.38
Potassium (meq/L)	4.4	±0.7	4.4	±0.7	4.4	±0.5	0.78
Calcium (mg/dl)	8.8	±0.6	8.8	±0.6	8.5	±0.7	0.09
Phosphorus (mg/dl)	4.2	±1.1	4.2	±1.1	4.2	±1.0	0.93
Albumin (g/dl)	3.8	±2.9	3.8	±2.9	3.6	±0.6	0.65
Cholesterol (mg/dl)	189	±56	189	±56	185	±60	0.76
Triglyceride (mg/dl)	165	±183	165	±184	135	±78	0.52
Fasting blood glucose (mg/dl)	136	±56	137	±56	130	±51	0.65

*BMI, body mass index; eGFR, estimated glomerular filtration rate using the Chronic Kidney Disease Epidemiology Collaboration (CKD-EPI) equation; BUN, blood urea nitrogen*.

Of 114 patients with TB infection, 50% patients had pulmonary TB, 28.1% patients had extra-pulmonary TB, and 21.9% patients had concomitant pulmonary and extra-pulmonary TB infection (Table 2). TB infections were found in one location in 74.6 % of patients, and 25.4% of patients had TB in more than one site. The most common extra-pulmonary TB infection sites in CKD patients were pleural (21.1%) and peritoneal infections (13.2%). Most TB infections were confirmed by the sputum culture (58.8%) followed by extra-pulmonary sites culture (12.3%), extra-pulmonary sites biopsy (7.9%), lung biopsy (7.0%), and pleural effusion ADA concentration more than 40 U/L (4.4%). Only 12.3% of TB cases at enrollment were diagnosed by the clinical presentation or image by the experts.

**Table 2:** Clinical characteristics of tuberculosis patients.

**Tuberculosis infection site**	***N* = 114 (%)**
Pulmonary infection	57 (50.0)
Concomitant infection	25 (21.9)
Extra-pulmonary infection	32 (28.1)
**Diagnosed method**	
Sputum culture	67 (58.8)
Lung biopsy	5 (4.4)
Extra-pulmonary site culture	14 (12.3)
Extrapulmonary site biopsy	9 (7.9)
Pleural effusion ADA > 40 U/L	5 (4.4)
By clinical presentation or image confirmed by expert	14 (12.3)

*ADA, Adenosine deaminase*.

**Figure 1:**
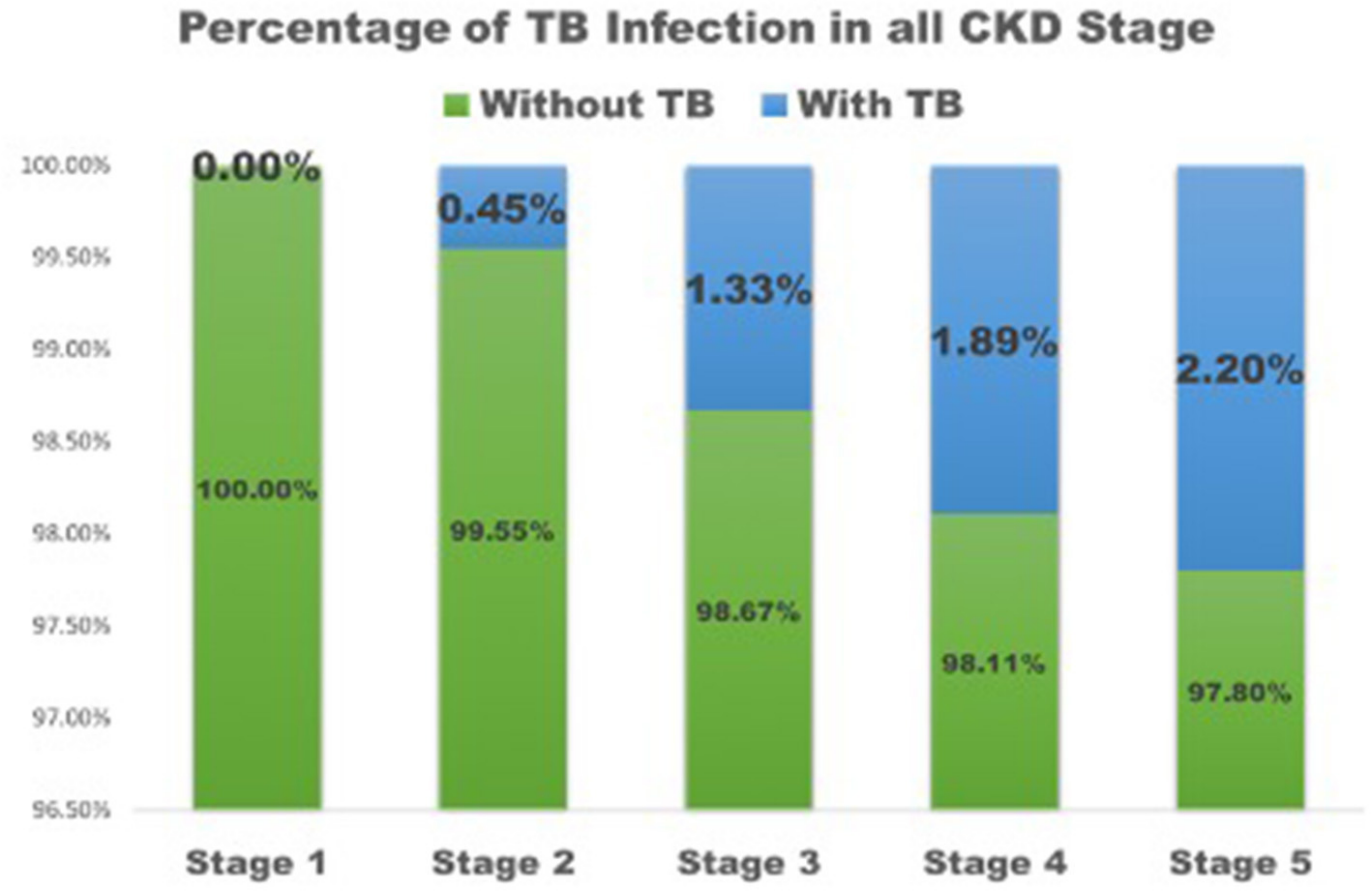
The percentage of TB infection in all CKD stage. TB, tuberculosis; CKD, Chronic kidney disease.

Patients with TB had lower eGFR (25 ± 18 ml/min/1.73 m^2^) compared to patients without TB (36 ± 26 ml/min/1.73 m^2^) (Table 1) and they had higher BUN (51 ± 26 mg/dl) than patients without TB (44 ± 31 mg/dl, *p* = 0.03). Patients who developed TB were older than those without TB (*p* < 0.01) and had a lower BMI (*p* < 0.001) than patients who did not have TB. The percentage of CKD stage 1 was higher in patients without TB, while the percentage of CKD stage 5 was higher in patients with TB (Figure 1). The causes of kidney disease were not different among patients with or without TB.

**Figure 2:**
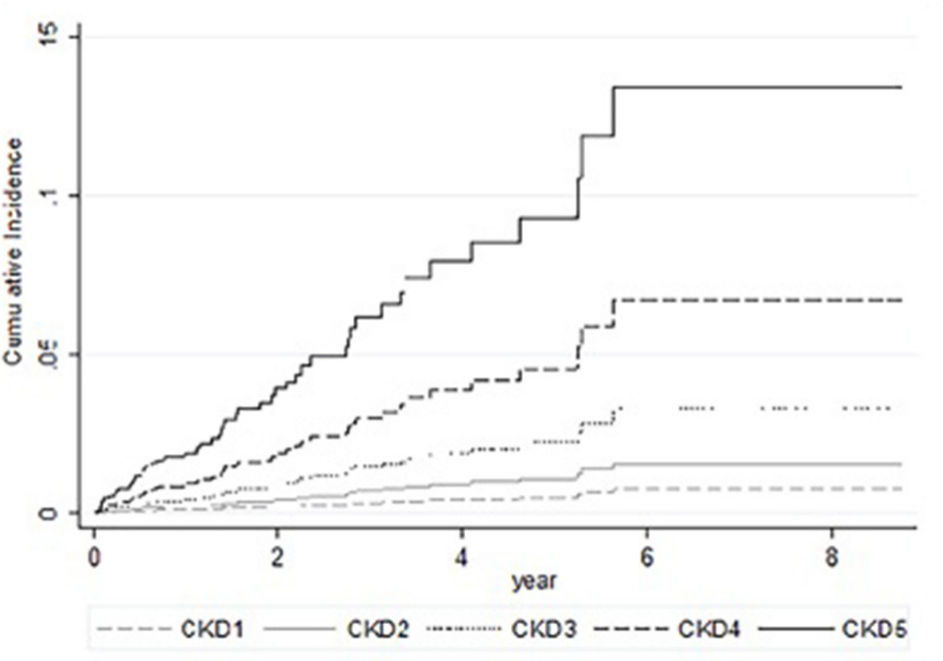
Cumulative incidence of tuberculosis in competing-risks analysis of patients with chronic kidney disease with adjustments for patients' age, gender, body mass index, diabetes, liver cirrhosis, serum calcium, and serum albumin.

In univariable competing-risk analysis (Table 3), higher eGFR was associated with a lower risk of TB infection with a SHR of 0.84 (95% CI: 0.79–0.90, *p* < 0.01) per 5 ml/min/1.73 m^2^. Among other factors, diabetes (*p* = 0.05), higher BUN (*p* < 0.01), higher creatinine (*p* < 0.01) were associated with the higher risk of TB infection while higher BMI (*p* = 0.04) and higher serum calcium (*p* = 0.02) were associated with lower risk of TB infection. Although patients' age, gender, smoking, liver cirrhosis, and serum albumin were not significantly different for the development of TB infection in univariable analysis, these factors were important risk factors of TB infection and were included in multivariable analysis (Table 4). Higher eGFR was independently associated a lower risk of TB infection with an adjusted SHR (aSHR) of 0.82 (95% CI: 0.72–0.94, *p* < 0.01). Higher BMI was also independently associated with a lower risk of TB infection (*p* = 0.01). The cumulative incidence of TB infection as per patients' CKD stages is shown in Figure 2. The risk of TB infection was highest in patients with CKD stage 5, followed by patients with CKD stage 4, stage 3, stage 2, and the risk were lowest in patients with CKD stage 1 (*p* < 0.01) with adjustment for patients' age, gender, BMI, diabetes, liver cirrhosis, calcium, and albumin.

**Table 3:** Subdistribution hazard ratios (SHRs) of possible confounders for tuberculosis in univariate competing-risks analysis.

**Possible confounders**	**SHR**	**95 %**	**CI**	***p***
eGFR (per 5 ml/min/1.73m^2^ higher)	0.84	0.79	0.90	<0.01
Age (per 10 additional years)	1.13	0.99	1.28	0.06
Male	1.05	0.70	1.58	0.81
Smoke	1.02	0.58	1.81	0.94
BMI (every 1 kg^2^/m^2^ higher)	0.94	0.88	0.99	0.04
Diabetes	1.48	1.00	2.01	0.05
Liver cirrhosis	2.22	0.97	5.07	0.06
Cardiovascular disease	1.58	0.77	3.25	0.21
Cancer	0.85	0.31	2.32	0.76
Hemoglobin (every 1 g/dl higher)	0.91	0.62	1.33	0.63
BUN (every 1 mg/dl higher)	1.02	1.01	1.02	<0.01
Creatinine (every 1 mg/dl higher)	1.20	1.15	1.25	<0.01
Uric acid (every 1 mg/dl higher)	1.21	0.89	1.64	0.22
Sodium (every 1 meq/L higher)	1.07	0.91	1.26	0.42
Potassium (every 1 meq/L higher)	1.45	0.80	2.62	0.22
Calcium (every 1 mg/dl higher)	0.58	0.37	0.91	0.02
Phosphorus (every 1 mg/dl higher)	1.16	0.74	1.82	0.52
Albumin (every 1 g/dl higher)	0.86	0.55	1.36	0.53
Cholesterol (every 1 mg/dl higher)	0.99	0.99	1.01	0.79
Triglyceride (every 1 mg/dl higher)	1.00	0.99	1.00	0.32
FBG (every 1 mg/dl higher)	1.00	0.99	1.01	0.97

*eGFR, estimated glomerular filtration rate using the Chronic Kidney Disease Epidemiology Collaboration (CKD-EPI) equation; BMI, body mass index; BUN, blood urea nitrogen; FBG, fasting blood glucose*.

**Table 4:** Adjusted subdistribution hazard ratios (aSHRs) of possible confounders for tuberculosis in multivariate competing-risks analysis.

**Possible confounders**	**aSHR**	**95%**	**CI**	***p***
eGFR (per 5 ml/min/1.37 m^2^ higher)	0.82	0.72	0.94	<0.01
Age (per 10 additional years)	0.98	0.79	1.23	0.89
Male	1.44	0.76	2.73	0.26
BMI (every 1 kg/m^2^ higher)	0.91	0.85	0.98	0.01
Smoking	0.84	0.35	2.01	0.70
Diabetes	1.53	0.84	2.80	0.17
Liver cirrhosis	2.72	0.96	7.70	0.06
Calcium (every 1 mg/dl higher)	0.59	0.29	1.20	0.15
Albumin (every 1 g/dl higher)	0.99	0.97	1.02	0.59

*eGFR, estimated glomerular filtration rate using the Chronic Kidney Disease Epidemiology Collaboration (CKD-EPI) equation; BMI, bod*.

## Discussion

Higher renal function was associated with a lower risk of TB infection with adjustments for confoundings for TB infection in the multivariate competing-risks analysis (Table 4) and the cumulative TB risk according to CKD stage (Figure 2). The competing-risks regression analysis was used because eGFR was also associated with all-cause mortality, and the risk of TB infection can be overestimated using Cox regression analysis (16). The increased risk of TB was also observed in the previous studies (7, 8). We further demonstrate the association between eGFR and TB risks. Factors associated with increase TB infection include prolonging corticosteroid use, diabetes (17, 18), human immunodeficiency virus infection, CKD (6), silicosis, malignancy, liver cirrhosis (19), and gastric surgery (20). Diabetes was associated with an increased risk of TB infection but was not significantly associated with TB infection risk in multivariable analysis (*p* = 0.17). Renal function may be a more powerful predictor of TB infection. The association of eGFR and TB infection may be explained by the impaired cellular immunity in CKD patients (21). CKD is associated with reduced vitamin D levels (22, 23), which plays a role in activating monocyte and macrophage (24–26). A low level of active form vitamin D level in these patients results in reduced monocyte and macrophage activation, thereby leading to the attenuated immune response. Thus, these patients are more susceptible to TB infection (27, 28). It may be worthwhile to study further if vitamin D replacement may decrease the risk of TB infection (29).

Smoking is one of the most common host-related factors associated with TB infection (30–32). However, we did not find a significant association between smoking and TB infection in our patients. The low prevalence of smoking may explain this. Malnutrition is another important host-related factor associated with TB infection (33–35). We found that CKD patients with lower BMI were associated with increased TB infection risk in multivariable analysis, but it did not mean malnutrition because the mean and standard deviation were within normal limits. The serum albumin level at enrollment was not significantly associated with TB infection in this study. The association between BMI and TB infection may be explained by chronic inflammation. Chronic inflammation is one of the non-traditional cardiovascular disease risks. Interlukin-6 is a marker for chronic inflammation and is negatively correlated to BMI (36). Further studies are needed to explore the interaction of interleukin-6, BMI, and TB infection.

50% of patients had pulmonary TB, and the other 50% had extra-pulmonary TB or concomitant TB infection. The percentage of extra-pulmonary infection plus concomitant infection was similar to the previous report (37) (48.4% of pulmonary TB; 37.7% of extra-pulmonary TB, and 13.9 % concomitant infection in maintenance dialysis patients). This distribution is different from that of the general population (82.3% of pulmonary TB, 10.6% of extra-pulmonary TB, and 7.1 % concomitant infection) (37). One of the unique characteristics of TB infection in dialysis patients is the high rate of extra-pulmonary infection, especially lymphadenitis (6, 37). In our report, the most common infections were pleural and peritoneal infections.

There were some potential limitations in this study. Information about steroid or biologics use was not recorded, and hence an association of steroid or biologics and TB infection could not be analyzed. It was a potential source bias. Some confoundings of TB infection were not recorded, such as alcohol consumption, drug dependence, migrants, unemployed, and homeless individuals. None of our patients had HIV infection. We are not able to analyze the association between TB infection and HIV. The generalizability of our findings is limited by the outpatient settings and CKD cohort of one hospital. Further studies of multiple institutions in different ethnic groups are needed to validate our findings.

## Conclusions

The risk of tuberculosis is negatively correlated with the renal function determined by using the estimated glomerular filtration rate, and this association is independent of patients' body mass index, age, and comorbidity in non-dialysis CKD patients. Physicians should pay more attention to the risk of tuberculosis infection in clinical practice, especially in patients with advanced kidney disease.

## Data Availability Statement

The original contributions presented in the study are included in the article/supplementary material, further inquiries can be directed to the corresponding author/s.

## Ethics Statement

The studies involving human participants were reviewed and approved by Internal review board approval (DMR 99-IRB-301). Written informed consent for participation was not required for this study in accordance with the national legislation and the institutional requirements.

## Author Contributions

C-HL, H-JC, D-TB, and C-YC were responsible for protocol design, data analysis, discussion, manuscript writing, and proofreading. W-CC, C-YT, T-CH, W-HH, C-TC, and C-CH were responsible for data collection. C-HL, D-TB, and C-YT were responsible for all work supervision and final manuscript approval. All authors contributed to the article and approved the submitted version.

## Conflict of Interest

The authors declare that the research was conducted in the absence of any commercial or financial relationships that could be construed as a potential conflict of interest.

## Publisher's Note

All claims expressed in this article are solely those of the authors and do not necessarily represent those of their affiliated organizations, or those of the publisher, the editors and the reviewers. Any product that may be evaluated in this article, or claim that may be made by its manufacturer, is not guaranteed or endorsed by the publisher.
